# Impact of the COVID-19 Pandemic on Teacher Quality of Life: A Longitudinal Study from before and during the Health Crisis

**DOI:** 10.3390/ijerph18073764

**Published:** 2021-04-04

**Authors:** Pablo A. Lizana, Gustavo Vega-Fernadez, Alejandro Gomez-Bruton, Bárbara Leyton, Lydia Lera

**Affiliations:** 1 Laboratory of Morphological Sciences, Instituto de Biología, Pontificia Universidad Católica de Valparaíso, Valparaíso 2373223, Chile; gustavo.vega@pucv.cl; 2 GENUD ‘‘Growth, Exercise, Nutrition and Development’’ Research Group, Faculty of Sport and Health Sciences, Universidad de Zaragoza, 50009 Zaragoza, Spain; bruton@unizar.es; 3 Public Nutrition Unit, Institute of Nutrition and Food Technology, University of Chile, Santiago 7830490, Chile; bleyton@inta.uchile.cl (B.L.); llera@inta.uchile.cl (L.L.); 4 Online Education, Keiser University, 1900 W Commercial Blvd, Fort Lauderdale, FL 33309, USA

**Keywords:** pandemic, COVID-19, school teachers, quality of life, mental health

## Abstract

Background: Prior to the COVID-19 pandemic, teachers were already reporting a low quality of life (QoL) perception, with a significant impact on mental and physical health due to various stress factors associated with work overload. The objective of this study was to evaluate the QoL impact on Chilean teachers before and during the COVID-19 pandemic. The analysis was performed following a longitudinal design on a sample of 63 Chilean teachers in pre-pandemic and COVID-19 pandemic timeframes. QoL perception, along with teachers’ sociodemographic data, was evaluated via the Short-Form 36 Health Survey (SF-36) questionnaire. Sociodemographic variables presented no significant variations in pre-pandemic and pandemic comparisons. QoL, however, showed a significant decrease during the pandemic compared to the pre-pandemic measurement (*p* < 0.01). In each gender, there were significant differences between pre-pandemic and pandemic timeframes, with a greater impact among women in the mental and physical component summary variables and seven of the eight QoL scales (*p* < 0.01). Between age categories, people under 45 presented significant differences (*p* < 0.05) between pre-pandemic and pandemic timeframes in all summary dimensions and measurements. In conclusion, Chilean teachers’ QoL perception has been affected by the COVID-19 pandemic. These findings could be related to work overload due to teleworking or feelings of uncertainty, loneliness, and fear that the pandemic and its associated confinements will worsen.

## 1. Introduction

Due to the rapid worldwide spread of the coronavirus (COVID-19), work organizations have had to adapt to public health measures regarding social distancing to reduce viral dissemination, forcing a massive shift towards teleworking [1]. In this context, teleworking is a factor that has been a major challenge for some professionals, with a lack of control over working hours and increased psychosocial risks associated with stress and work overload [2,3]. Furthermore, teleworking has had a significant impact on professional and personal life (work–life balance), producing physical and mental exhaustion and burnout [4]. Additionally, the increase in digital technologies at work has increased stress in workers (techno-stress), which is associated with significant psychosocial demands [5]. The concept of techno-stress includes the adverse effects caused by technology on people’s behaviors and physiology [6]. In this context, as psychosomatic consequences are recognized, over time, teachers may develop high levels of burnout [7]. Thus, in Chile, techno-stress (techno-anxiety, techno-fatigue, or both conditions) had already been reported in teachers who incorporated computer and communication technologies into their practices before the COVID-19 pandemic [8]. In this context, techno-stress in teachers may be caused by the introduction of technology to the classroom and a lack of adaptation to the technological environment [9]. Due to the global crisis caused by COVID-19, the change from face-to-face to an online format may affect teachers’ mental health due to the short adaptation period. 

The COVID-19 pandemic created rapid global change that affected the teaching world. In many countries, the situation was approached with strict confinement by closing educational establishments [10] and obligating teachers to swiftly adapt to distance learning [11]. In Chile, 3 March 2020 saw the first case of the SARS-CoV-2 virus, which can cause severe acute respiratory syndrome [12]. Throughout the Chilean territory, on 16 March, churches, schools, and gymnasiums were closed by decree, followed by the closure of international borders. From the beginning, plans for social isolation were implemented, with dynamic quarantines between Chilean regions that were eventually extended through various regional capitals and cities showing higher infection rates. Some cities reached 120 days of total quarantine. The health crisis outlook is a reflection of the critical scenario in many developed and developing countries, where the psychosocial impact has been reported as significantly high throughout the population [13]. This is a context in which differences in the quality-of-life (QoL) perception of the population could emerge, especially among the teacher labor pool, which, prior to the COVID-19 pandemic, was already reporting diminished QoL perception associated with various factors [14,15].

Teachers have experienced an important change in their work format during the global health emergency [16]. Prior to the pandemic, these professionals were widely studied, and research showed a high work overload that led to work burnout [17,18]. Some reports indicate that stressors present among teachers include poor working conditions, difficulties with students and families, and work organizational factors [19]. In the educational workforce, teachers must organize their work and allot extra hours for tending to parents and guardians, preparing materials, and planning, which is mostly done at home [20]. On top of this, teachers have reported various epidemic problems, such as high rates of chronic non-transmissible diseases associated with QoL deterioration [15], high prevalence of obesity and low physical activity (associated with post-work fatigue and very late work hours) [18], high rates of musculoskeletal disorders, burnout syndrome, depression, and anxiety [21,22]. Additionally, these problems are exacerbated among females [23], a highly important sociodemographic factor given the high proportion of women in this profession [20,24]; age is also a significant factor associated with greater mental and physical deterioration [14,15]. All of this takes place within a context of work overload multiplied by teleworking and other factors that result in physical and mental QoL deterioration [19,25]. Therefore, the objective of this study was to compare health-related QoL in teachers before and during the COVID-19 pandemic.

## 2. Materials and Methods

### 2.1. Participants

During the COVID-19 pandemic, 155 random teachers were contacted via e-mail, all of whom had participated in the Chilean National Fund for Scientific and Technological Research project (FONDECYT-ANID 11170716) before the pandemic. Ninety-two teachers were excluded due to incomplete data, leaving a sample of 63 teachers (from 13 schools; 46.15% from northern Chile, 23.08% from central Chile, and 30.77% from southern Chile), who participated in both evaluations and satisfied the longitudinal design of this study.

### 2.2. Instruments

All sociodemographic data on participants were gathered through surveys. To evaluate teachers’ QoL, the Short-Form 36 Health Survey (SF-36) instrument, which was developed in the USA to evaluate QoL perception related to health in adults, was applied [26]. This questionnaire was adapted syntactically and semantically to Chilean idiosyncrasy and applied to a representative sample of the Chilean adult population [27]. The instrument consists of 36 Likert-type personal appraisal questions grouped into eight scales: physical function, role limitations due to physical problems, bodily pain, general health perceptions, vitality, social functioning, role limitations due to emotional problems, and mental health. These scales are also grouped into two summary measurements: a physical component summary (PCS) and a mental component summary (MCS). Participants’ scores for each scale and component are transformed into a scale of 0–100, followed by calculating z-score and t-score values for each scale and summary measurements in the mental and physical component summaries with an internationally standardized method [28].

To calculate the scores for each scale and component, the method using standardized scores in the USA was applied. For the general population, the scores of each scale were obtained with the following transformation:

T = z-score × 10 + 50

The values of each component were obtained with the following:

SF-36 PCS = Σ (z-score of each scale × respective physical factor coefficient) × 10 + 50

SF-36 MCS = Σ (z-score of each scale × respective mental factor coefficient) × 10 + 50

This transformation, with a mean of 50 and an SD of 10, allows the results to be directly interpretable. Thus, scores above 50 indicate a better QoL and scores below 50 indicate a worse QoL than the mean of the reference population. Regarding the reliability of the SF-36 scale, Cronbach’s alpha coefficient was high and homogeneous (α = 0.85) for each of the eight SF-36 scales. Factor analysis yielded two factors; the first and second factors accounted for 77% and 32% of the total variance and 90% and 38% of the reliable variance, respectively. The data met the Kaiser–Meyer–Olkin measure (0.85) with small values, meaning that, overall, the variables had too little in common to warrant a factor analysis. Pearson’s correlation coefficients of the eight scale scores with MCS indicated a high correlation for vitality, social functioning, role limitations due to emotional problems, and mental health, ranging from r = 0.92 (mental health) to r = 0.78 (social functioning). On the other hand, the medium and highest correlations for PCS were obtained for the scales of physical function, role limitations due to physical problems, bodily pain, and general health perceptions, ranging from r = 0.74 (body pain) to r = 0.58 (general health perceptions), as shown in Appendix A, Table A1, Table A2, Table A3 and Table A4.

### 2.3. Procedure 

Teachers’ QoL was evaluated in two stages: pre-pandemic and pandemic. The evaluation of pre-pandemic subjects included teachers from the FONDECYT-ANID 11170716 study who were interviewed in person between October 2018 and October 2019; during the pandemic, contact was reestablished with teachers via email between July and October 2020. The teachers who agreed to participate answered a Spanish survey via an online survey platform named SurveyMonkey (SurveyMonkey, San Mateo, CA, USA). 

No monetary compensation was given for completing the questionnaire. The study met Helsinki Declaration guidelines [29]. Participating teachers signed informed voluntary consent forms prior to collecting their background information (sociodemographic data and SF-36 questionnaire), which explicitly said that all personal results are strictly confidential. All procedures for this study were approved by the bioethics committee of the Pontificia Universidad Católica de Valparaíso.

### 2.4. Statistical Analysis

Data were analyzed with STATA 16 software for Windows. Descriptive statistics were analyzed using measures with standard deviations (SDs) for continuous variables and frequencies with percentages for categorical variables (*n*, %). Sociodemographic variables were compared between genders in the pre-pandemic and pandemic timeframes. QoL was also evaluated by comparing each scale and summary measure between the pre-pandemic and pandemic periods for each of the two teacher age groups (≤44 years old and ≥45 years old). Age categories were obtained from the Chilean National Health Survey 2009/2010 [30], and QoL (according to all scales) was compared between pre-pandemic and pandemic periods in males and females. Specific tests were used for comparing medians (*t*-test for related samples or their non-parametric equivalent, Wilcoxon) according to results of the Shapiro–Wilk normality test, and the chi-squared and Fisher’s exact association tests were used to analyze categorical variables.

## 3. Results

### 3.1. Participant Characteristics 

Of the 63 study participants, 71% were women (*n* = 45). In Table 1, we present the sociodemographic characteristics analyzed between genders in pre-pandemic and pandemic periods, with all variables showing no significant differences between the two evaluation periods. 

### 3.2. Quality of Life 

In Table 2, we can observe a comparison of scores on each of the eight scales and the two summary measurements on the SF-36 survey in the pre-pandemic and pandemic timeframes for the total sample of individuals. Participants in the pre-pandemic period presented higher scores on QoL perception in all dimensions in comparison with measurements taken during the pandemic. Comparisons of all QoL dimensions in pre-pandemic and pandemic timeframes were all significant (*p* < 0.01). During the pandemic, the dimensions with the lowest scores were social functioning (35.251 ± 12.826), mental health (36.868 ± 10.783), and the mental component summary (34.959 ± 10.3). 

Table 2 also shows a comparison of the scores on each of the eight scales and the two summary measurements on the SF-36 survey in the pre-pandemic and pandemic timeframes by gender. The results show differences between the two periods for men and women. In men, there were statistically significant differences in the dimensions of role limitations due to physical problems, general health perceptions, social functioning, and mental health (*p* < 0.05). However, among women, every dimension and summary measurement presented significant differences (all *p* < 0.05).

Table 3 shows the differences in pre-pandemic versus pandemic scores on all QoL dimensions for each teacher age category (≤44 and ≥45). Teachers aged ≤44 years showed a significant decrease (*p* < 0.05) between pre-pandemic and pandemic timeframes in all the measured variables, except for role limitations due to emotional problems (*p* = 0.190). The second age category (≥45 years) only showed a significant decrease in QoL for role limitations due to physical problems, vitality, role limitations due to emotional problems, mental health dimensions, and the mental component summary (*p* < 0.05).

## 4. Discussion

The purpose of the present study was to compare Chilean teachers’ QoL in a longitudinal study between pre-pandemic and pandemic timeframes. The principal results indicate that Chilean teachers presented decreased scores on their health-related QoL perception before the pandemic. This background has also been observed in other regions of the world [22], as well as among Chilean teachers, who reported a significant association between low QoL perception and the mental component summary dimension among younger teachers (≤44 years vs. ≥45 years) [15]. Additionally, during the pandemic, the scores dropped significantly; this may be due to the impact of teleworking on teachers’ health, as reported in other groups of workers, who indicated that it was a principal factor impacting psychosocial health and physical burnout due to stress and work exhaustion among employees [2,3,4]. However, more studies are required to evaluate the effect of telework on teachers during the COVID-19 pandemic, as these results could also be due to other factors not explored in the present study, such as a decrease in social relations, domestic confinement, and a reduction in physical activity levels [31].

Our results in the pandemic context indicate that QoL impact occurred specifically among women in the ≤44 age category. These findings coincide with recent studies in an Italian population, which showed a significantly lower psychological wellbeing among women, people under 50, and individuals with health risk factors [32]. Additionally, similar results were reported from Austria, where women and young adults (<35 years), along with the unemployed and poor, presented mental health problems all related to an increase in depression and decrease in QoL [33]. The same group of researchers saw similar problems emerge in the population of the United Kingdom: adults under 35, women, and unemployed people were the most affected by confinement in terms of mental health [34]. These results also align with those reported from Iran, where women and young adults had the most anxiety about COVID-19 [35]. In China, during the initial COVID-19 outbreak, women were also seen to have greater psychological impacts and higher levels of stress, anxiety, and depression [36]. In Greece, female teachers had greater feelings of fear and depression at the beginning of the pandemic [37], and in China, female teachers reported higher anxiety than male teachers [38]. In this regard, Riecher-Rössler (2020) stated that in spite of important evidence that shows differences in mental disorders by sex and gender, there is still little real comprehension of these differences’ causes [39]. One explanation for the greater QoL impact among women during the pandemic could be the heavy load of self-assumed or socially imposed home responsibilities, even among women professionals [40]. Recently, in Chile, it was reported that female teachers experienced significant work exhaustion and lower engagement compared to their male work peers, regardless of whether there were children in their home, results that can be complemented with other pre-pandemic studies in Chile, which reported that working-age women had a higher probability of suffering stress than men [41]. On top of these results, various researchers have reported that female Latin American teachers do more hours of housework than male teachers [15,20]. This study, because it is longitudinal, could indicate that the COVID-19 confinement phenomenon is what primarily impacts QoL perception, especially among female teachers in the physical and mental component summaries. There are similar reports from studies in other populations, such as Greek university students, where confinement caused QoL deterioration, tripled depression cases, and increased suicidal ideation eightfold [42]; studies in Russia maintained that fear and loneliness from confinement could have negative consequences for the mental and physical health of people [43], and results obtained in Spanish teachers during the pandemic revealed that they have experienced higher levels of distress due to the workload generated during the lockdown [44].

Longitudinal studies on COVID-19 among teachers are scarce. However, Sokal et al. (2020) conducted a study surveying Canadian teachers at two points at the beginning of the COVID-19 pandemic. Their results indicated increasing burnout and cynicism, along with teachers’ emotional and cognitive attitudes towards change becoming increasingly negative. Recently, in Chile, a survey on teacher burnout was conducted, which indicated decreased engagement and increased work exhaustion in individuals. These results can be complemented by the results of this study based on the impact on teachers’ mental and physical health [45]. One relevant aspect of the study on engagement and work burnout among Chilean teachers is that it compared its results with workers in various occupations and professions before and during the pandemic, revealing that Chilean teachers had less engagement and more work exhaustion than other labor groups [45]. The observation proposed by Foundation Chile (2020) about pre-pandemic work exhaustion among Chilean teachers can be confirmed through the present results, which show that physical function and role limitation (due to emotional problems) dimensions alone are over 50 points, corresponding to a welfare cutoff point. These results suggest that pre-pandemic teachers were already suffering from mental and physical wellbeing levels below those of other professionals, and that since the pandemic, these figures have dropped significantly. These results support those described in the present article: when asked about the possible worsening of the pandemic, teachers’ mental and physical deterioration is ever higher. Recent observations indicate that teachers show a high prevalence of anxiety, depression, and stress in places where face-to-face classes have returned [46]. 

The presented study results suggest a substantial impact on QoL due to working conditions during the COVID-19 pandemic, with a significant effect on women and the youngest age group. Furthermore, these results suggest that the mental and physical recovery of Chilean teachers will be challenging. Future strategies should be focused on reducing the physical and psychological impacts generated by COVID-19 confinement. 

### Limitations

Participants’ replies were self-reported, which is considered a limitation. However, by applying the same instrument on two widely separated occasions, comparisons were made in different contexts, strengthening the results. On the other hand, as a longitudinal study, this research also has the strength of understanding how the pandemic phenomenon impacts teachers. Some variables that could affect the main outcomes, such as physical activity levels or social relations, were not registered in the present study and could partially explain some of the findings. Finally, an important limitation is the sample size, so the results should be interpreted with caution, and further studies will be necessary with larger samples. However, the sample demographics are comparable to those in previous studies. The percentage of female teachers is very similar to other studies and the Chilean Ministry of Education’s national report (≥70%) [15,16,24,47]. Furthermore, age also maintains a similar proportion to that reported by the Chilean Ministry of Education. The majority of teachers (>60%) are 44 years of age or less [47]. Concerning the educational establishments where the teachers work, there was a representation from the northern, central, and southern regions of the country, representing Chile’s macro-zones.

## 5. Conclusions

In the sample of teachers studied, low QoL scores were observed before the COVID-19 pandemic, and they decreased significantly during the pandemic, especially among women and individuals under 45 years old. These findings confirm the deterioration of teachers’ QoL during the pandemic. This study reports some negative impacts of the COVID-19 pandemic on teachers’ mental and physical health. The present results should serve as a resource for future interventions among teachers to help improve their QoL. 

## Figures and Tables

**Table 1:** Sociodemographic characteristics of Chilean teachers analyzed by gender before and during the COVID-19 pandemic (*n* = 63).

	Pre-Pandemic			Pandemic		
	Male	Female	*p* Value	Male	Female	*p* Value
Age (years) ^d^	41.2 ± 13.3	37.3 ± 10.7	0.360 ^a^	42.6 ± 13.4	38.9 ± 10.9	0.398 ^a^
≤44 ^e^	10 (55.6)	33 (73.33)	0.232 ^b^	10 (58.82)	31 (67.39)	0.562 ^b^
≥45 ^e^	8 (44.4)	12 (26.67)		7 (41.18)	151 (32.61)	
Marital status ^e^						
Single	16 (88.89)	41 (91.11)	0.07 ^b^	16 (88.89)	41 (91.11)	0.07 ^b^
Married/partnered	2 (11.11)	4 (8.89)		2 (11.11)	4 (8.89)	
Experience work (years) ^d^				15.944 ± 13.50	13.355 ± 10.81	0.731 ^a^
Type of contract ^e^						
Fixed-term	18 (100)	45 (100)	-	18 (100)	45 (100)	-
Type of school ^e^						
Public (state)	2 (11.11)	8 (17.78)	0.473 ^c^	3 (16.67)	10 (22.22)	0.546 ^c^
Private (subsidized)	12 (66.67)	22 (48.89)		11 (61.11)	20 (44.44)	
Private (non-subsidized)	4 (22.22)	15 (33.33)		4 (22.22)	15 (33.33)	
Domestic work ^e,f^						
<15 h	18 (28.57)	45 (71.43)	-	13 (72.22)	36 (80.00)	0.502 ^b^
>15 h	-	-		5 (27.78)	9 (20.00)	

<15 h and >15 h, domestic work in hours; *p* < 0.05. ^a^ Wilcoxon test, ^b^ Fisher’s exact test, ^c^ chi-squared test. ^d^ Data are expressed as mean and standard deviation. ^e^ Data are expressed as frequency (percentage). ^f^ Performing household chores, either your own or someone else’s, without payment (e.g., cooking, cleaning, shopping, laundry, ironing, child care, etc.).

**Table 2:** Comparison of Short-Form 36 Health Survey (SF-36) measurements of 8 scales and quality of life (QoL) summary measurements evaluated in pre-pandemic and pandemic timeframes in the total sample and each gender.

	Total Sample (*n* = 63)			Male (*n* = 18)			Female (*n* = 45)		
	Pre-Pandemic	Pandemic		Pre-Pandemic	Pandemic		Pre-Pandemic	Pandemic	
QoL	Mean ± SD	Mean ± SD	*p*	Mean ± SD	Mean ± SD	*p*	Mean ± SD	Mean ± SD	*p*
Physical function	51.223 ± 6.72	47.930 ± 8.17	0.002 ^b^	48.762 ± 9.29	46.942 ± 8.78	0.457 ^b^	52.208 ± 5.18	48.325 ± 7.99	<0.001 ^b^
Role limitations due to physical problems	47.494 ± 6.71	44.009 ± 7.67	0.007 ^a^	49.090 ± 6.47	44.056 ± 7.75	0.005 ^b^	50.672 ± 4.79	45.206 ± 7.02	<0.001 ^b^
Bodily pain	44.682 ± 8.56	38.543 ± 9.96	<0.001 ^a^	46.254 ± 9.97	42.061 ± 10.34	0.224 ^a^	44.053 ± 7.96	37.135 ± 9.56	<0.001 ^a^
General health perceptions	47.997 ± 9.51	43.236 ± 10.37	<0.001 ^b^	49.178 ± 8.79	41.603 ± 10.70	0.026 ^a^	47.525 ± 9.83	43.889 ± 10.29	0.004 ^b^
Vitality	46.795 ± 7.73	40.187 ± 8.21	<0.001 ^a^	46.533 ± 7.55	43.704 ± 7.89	0.114 ^a^	45.967 ± 9.69	38.779 ± 7.98	<0.001 ^a^
Social functioning	41.99 ± 11.43	35.251 ± 12.82	0.003 ^a^	43.675 ± 10.36	32.812 ± 14.61	0.015 ^a^	41.316 ± 11.83	36.226 ± 12.08	0.047 ^a^
Role limitations due to emotional problems	47.494 ± 6.72	44.009 ± 7.67	0.008 ^a^	47.253 ± 7.69	44.449 ± 8.66	0.311 ^a^	47.589 ± 6.38	43.832 ± 7.33	0.011 ^a^
Mental health	45.769 ± 8.86	36.868 ± 10.78	<0.001 ^a^	46.386 ± 9.66	38.520 ± 11.47	0.033 ^a^	44.350 ± 11.48	36.207 ± 10.55	<0.001 ^a^
Physical component summary	47.33 ± 6.23	43.414 ± 6.94	0.001 ^b^	46.573 ± 7.17	43.086 ± 7.74	0.170 ^a^	47.633 ± 5.87	43.545 ± 6.67	0.002 ^a^
Mental component summary	42.074 ± 9.68	34.959 ± 10.30	<0.001 ^a^	43.194 ± 9.96	36.137 ± 11.04	0.052 ^a^	41.626 ± 9.65	34.489 ± 10.07	<0.001 ^a^

*p* < 0.05 ^a^ *t*-test, ^b^ Wilcoxon’s text.

**Table 3:** Comparison of 8 SF-36 QoL scales and two summary measurements between pre-pandemic and pandemic timeframes for each age group ≤44 and ≥45 years (*n* = 63).

	≤44 (*n* = 41)			≥45 (*n* = 22)		
	Pre-Pandemic	Pandemic		Pre-Pandemic	Pandemic	
QoL	Mean ± SD	Mean ± SD	*p*	Mean ± SD	Mean ± SD	*p*
Physical function	52.828 ± 4.45	48.993 ± 7.49	0.007 ^b^	48.232 ± 9.01	46.949 ± 9.17	0.134 ^b^
Role limitations due to physical problems	50.150 ± 5.47	44.918 ± 7.09	<0.001 ^b^	50.351 ± 5.16	44.803 ± 7.54	0.007 ^a^
Bodily pain	44.298 ± 8.85	38.431 ± 10.74	0.009 ^a^	45.397 ± 8.06	38.750 ± 8.57	0.011 ^a^
General health perceptions	47.995 ± 9.82	42.795 ± 11.05	0.002 ^b^	48.001 ± 9.13	44.057 ± 9.19	0.161 ^a^
Vitality	45.579 ± 7.78	39.078 ± 8.09	<0.001 ^a^	49.059 ± 7.26	42.252 ± 8.19	0.006 ^a^
Social functioning	41.525 ± 10.81	35.394 ± 13.01	0.022 ^b^	42.856 ± 12.63	34.985 ± 12.78	0.046 ^a^
Role limitations due to emotional problems	46.186 ± 6.97	44.094 ± 7.37	0.190 ^a^	49.929 ± 5.57	43.850 ± 8.36	0.007 ^a^
Mental health	44.738 ± 8.83	35.868 ± 10.50	<0.001 ^a^	47.690 ± 8.79	38.731 ± 11.29	0.005 ^a^
Physical component summary	48.311 ± 5.86	43.905 ± 7.26	0.003 ^a^	45.502 ± 6.61	42.499 ± 6.34	0.030 ^b^
Mental component summary	40.227 ± 9.78	34.056 ± 10.11	0.006 ^a^	45.432 ± 8.77	36.641 ± 10.67	0.005 ^a^

≤44 and ≥45, age categories (years) *p* < 0.05, ^a^ *t*-test, ^b^ Wilcoxon test.

## Data Availability

The datasets generated and/or analyzed during the current study are available from the corresponding author on reasonable request.

## Appendix A

**Table A1:** Factor analysis SF-36 for Chilean teachers. Rotated factor loadings and variance explained.

	Factor 1a MCS	Factor 2a PCS
Physical function	0.1078	**0.4363**
Role limitations due to physical problems	0.4573	**0.5451**
Bodily pain	0.1993	**0.5618**
General health perceptions	**0.5300**	**0.4785**
Vitality	**0.7813**	0.2193
Social functioning	**0.6807**	0.3027
Role limitations due to emotional problems	**0.7421**	0.2127
Mental health	**0.8719**	0.1026
Variance explained		
Total (%)	76.9	32.23
Reliable ^b^ (%)	90.19	37.92

Results of a factor analysis (two factors retained) using the principal factor method with a varimax rotation. MCS and PCS: Mental and Physical health summaries. Bold values indicate variables included in the factors: MCS: loading > 0.52; PCS: loading > 0.34. b Reliable variance = total variance explained divided by the internal reliability (Cronbach’s a) of the scale.

**Table A2:** Kaiser-Meyer-Olkin (KMO) measure of sampling adequacy.

	KMO
Physical function	0.7993
Role limitations due to physical problems	0.8600
Bodily pain	0.7399
General health perceptions	0.8944
Vitality	0.8472
Social functioning	0.9056
Role limitations due to emotional problems	0.8751
Mental health	0.7966
Overall	0.8487

Kaiser-Meyer-Olkin (KMO) takes values between 0 and 1, with small values meaning that overall the variables have too little in common to warrant a factor analysis (0.00 to 0.49 unacceptable; 0.50 to 0.59 miserable; 0.60 to 0.69 mediocre; 0.70 to 0.79 middling; 0.80 to 0.89 meritorious; 0.90 to 1.00 marvelous).

**Table A3:** The internal consistency for each of the eight SF-36 scales (Cronbach’s alpha).

	Item-Test Correlation	Item-Rest Correlation	Average Interitem Correlation	Alpha
Physical function	0.4532	0.2948	0.4859	0.8687
Role limitations due to physical problems	0.7371	0.6373	0.4100	0.8295
Bodily pain	0.5622	0.4212	0.4567	0.8548
General health perceptions	0.7559	0.6616	0.4050	0.8265
Vitality	0.7859	0.7009	0.3970	0.8217
Social functioning	0.7659	0.6747	0.4023	0.8249
Role limitations due to emotional problems	0.7668	0.6758	0.4021	0.8248
Mental health	0.7850	0.6997	0.3972	0.8218
Test scale				0.8526

**Table A4:** Correlation between the summary measures of the SF-36 and each of the eight dimensions.

	MCS	PCS
Physical function	0.0007	**0.6924**
Role limitations due to physical problems	0.4372	**0.5729**
Bodily pain	0.1471	**0.7419**
General health perceptions	0.5199	**0.5837**
Vitality	**0.8020**	0.2134
Social functioning	**0.7826**	0.2555
Role limitations due to emotional problems	**0.8111**	0.1133
Mental health	**0.9179**	0.0512

MCS and PCS: Mental and Physical health summaries. Bold values indicate the highest correlations for each factor.
